# Point-of-care screening for syphilis and HIV in the borderlands: challenges in implementation in the Brazilian Amazon

**DOI:** 10.1186/s12913-015-1155-y

**Published:** 2015-11-05

**Authors:** Carole Zen Ruffinen, Meritxell Sabidó, Ximena Pamela Díaz-Bermúdez, Marcus Lacerda, David Mabey, Rosanna W. Peeling, Adele Schwartz Benzaken

**Affiliations:** Swiss Tropical and Public Health Institute, University of Basel, Basel, Switzerland; Fundação de Medicina Tropical Doutor Heitor Vieira Dourado (FMT-HVD), Avenida Pedro Teixeira 25, CEP: 69040-000 Manaus, Amazonas State Brazil; TransLab. Department of Medical Sciences, Faculty of Medicine, Universitat de Girona, Catalunya, Spain; Departamento de Saúde Coletiva, Universidade de Brasília, Brasília, Brazil; Department of Clinical Research, London School of Hygiene and Tropical Medicine, London, UK; Departamento de DST/Aids, Fundação Alfredo da Mata, Manaus, Amazonas State Brazil; Departamento de IST, Aids e Hepatites Virais, Secretaria de Vigilância em Saúde, Ministério da Saúde, Brasília, Brazil

**Keywords:** Syphilis, HIV, Point-of-care testing, Implementation, Amazon

## Abstract

**Background:**

Point-of-care (POC) screening for HIV and syphilis using rapid testing was implemented in indigenous communities in the triple-border area of the Brazilian Amazon. We describe the context of the early introduction of POC screening, explore hindering and enabling factors for POC implementation, and recommend strategies for feasible, viable, and sustainable syphilis and HIV screening interventions.

**Methods:**

This was a qualitative study based on grounded theory methodology. Data were collected using in-depth interviews, semi-structured questionnaires, and field observations and were analysed using the framework approach. Qualitative information was complemented by quantitative data for descriptive purposes.

**Results:**

An overall high score for vulnerability to acquiring HIV and syphilis was observed among the indigenous communities. Health professionals reported satisfactory rapid testing acceptance, although concerns were raised about the pain of the fingerprick. Counselling-related challenges included ensuring the accuracy of translations, collaborating with translators and communicating positive test results. Over 3 months, 86.7 % of the syphilis-positive individuals began treatment, and all of them notified their partners. Accessibility, measured as travel time via the local transportation network, was a barrier to health care access. A lack of gasoline for boats and other transportation was also a hindering factor at all levels of implementation.

**Conclusions:**

The recommendations address the preparation phase at the coordination level as well as at the training level. Tools such as strengths, weaknesses, opportunities, and threats (SWOT) analyses; checklists; context-adapted protocols; and fact sheets are very simple methods to facilitate implementation. The findings of this study are important because they may inform the implementation of new health technologies in low-resource national disease control programmes in remote communities.

## Background

The Brazilian Amazon region is characterised by scattered and isolated communities, a fragile physical infrastructure, and a shortage and high rotation of health staff, all of which hinder efforts to implement point-of-care (POC) testing for syphilis and HIV. The prevalences of syphilis and HIV in the indigenous population living in this region are relatively low, at 1.6 and 0.1 %, respectively [1]. However, this population is subject to a high vulnerability to acquiring both infections due to the structural conditions of living and the deep epidemiological transition experienced in the indigenous context, both of which promote the spread of HIV and other STIs [2]. In Brazil, rapid testing for syphilis and HIV at the POC could facilitate early access to diagnoses and adequate treatments [3], thus accelerating efforts to eliminate congenital syphilis [4] and facilitating the widespread adoption of the HIV test-and-treat strategy [5].

From 2008 to 2010, a consortium of institutions introduced community-based POC screenings for syphilis and HIV among remote indigenous groups in the Brazilian Amazon. Screenings were not available previously, and many cases of both infections had remained undiagnosed [6]. This initiative provided an opportunity for all sexually active individuals in the states of Amazonas and Roraima to access testing. Screening was offered through nine Special Indigenous Health Districts (DSEIs). The SD Bioline Syphilis 3.0 rapid test (Standard Diagnostics, Kyonggi-do, Korea) was used for syphilis diagnoses, and the DPP HIV 1/2 rapid test from Bio-Manguinhos (Oswaldo Cruz Foundation, Rio de Janeiro, Brazil) was used for HIV diagnoses. The current study was conducted in the Alto Solimões DSEI, which is located in northern Brazil at the triple border with Colombia and Peru, during the first three months of the implementation process for community-based POC screening for HIV and syphilis.

The study objectives were to describe the context of the early introduction of POC screening via rapid testing into the existing indigenous health care system, to evaluate the performance of the health service in POC screening, and to delineate the factors that hinder and facilitate this implementation in practice.

**Fig. 1:**
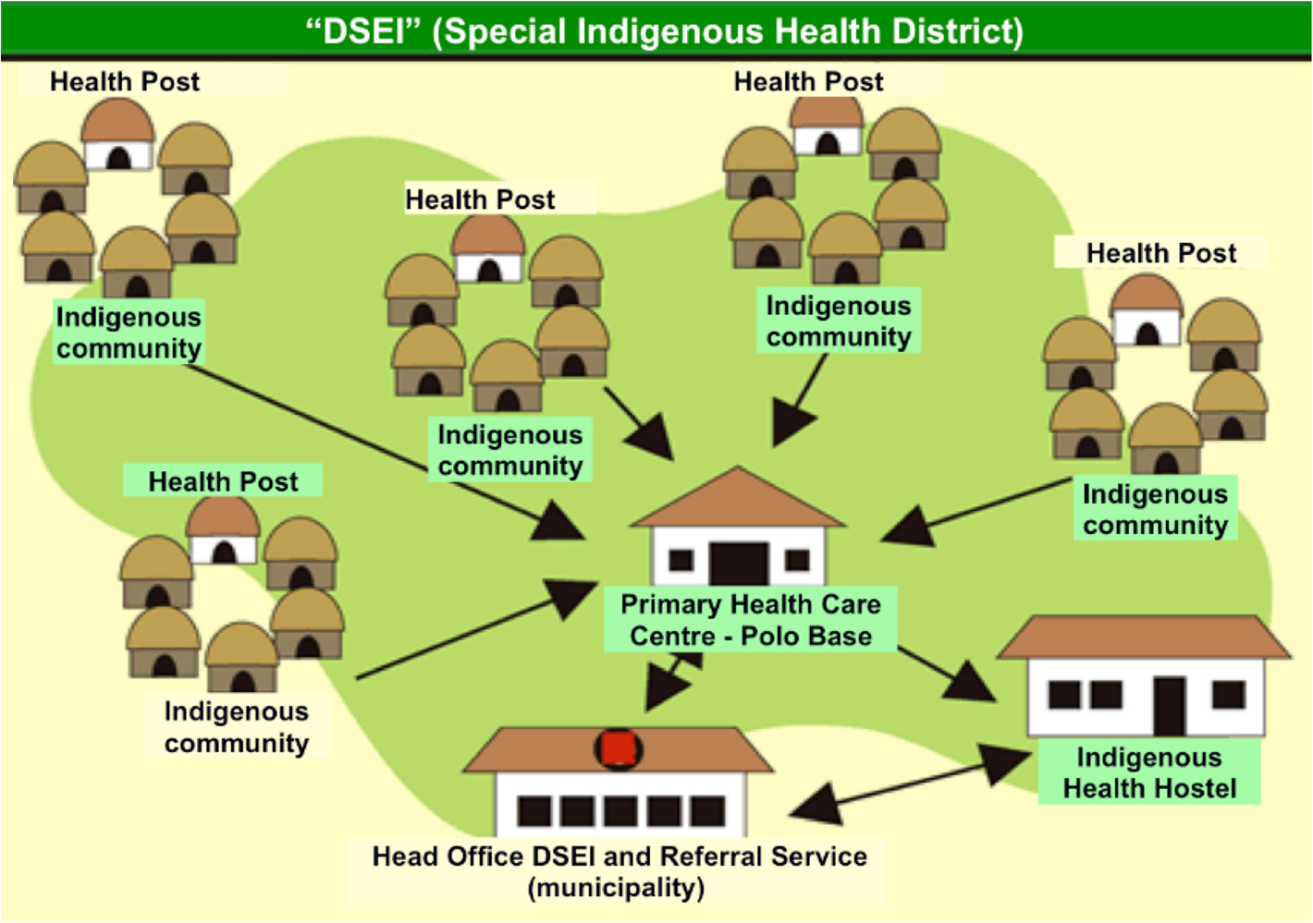
Structure of the DSEI and its health care model

The indigenous health care system is a special branch of Brazil’s publicly funded universal health care system [7]. DSEI boundaries are defined by ethno-cultural diversity rather than by geographical criteria and are autonomous and decentralised units that provide health care at different levels [8] (Fig. 1). At the central level, each DSEI has a head office, which is generally located in the area’s main municipality or city. A DSEI also represents the indigenous health authority and is responsible for administrative tasks and referral health services. In addition, basic primary health care units called polo bases and health posts are located at the peripheral level within the villages.

## Methods

### Study design

The study used a combined quantitative and qualitative approach to identify the complexity of implementing health actions that focus on indigenous people in a large territory. We targeted key informants and health professionals involved in screening and indigenous people from the different territories at the polo base level.

The triangulation approach let us explore a vast diversity of information by complementing a qualitative thematic framework with quantitative data. The study was performed in three different, though connected, stages, which allowed us to collect data in one phase that would lead to the next phase, thus gradually building a definitive framework until the findings reached saturation.

In the first phase, in-depth interviews with two programme coordinators and a medical doctor with extensive experience with the DSEI were conducted to collect descriptive data and to map the structures and operations of the local health services. These key actors also participated in a strengths, weaknesses, opportunities, and threats (SWOT) analysis [9] (Table 1) of the main project. In addition, semi-structured field observations (FO) [10] focusing on infrastructure, storage, testing, counselling, and data collection were carried out at one polo base. All of this information assisted in the design of a baseline questionnaire.

**Table 1:** Strengths, weaknesses, opportunities, and threats (SWOT) analysis of the screening programme for HIV and syphilis in Alto Solimões

Strengths	Weaknesses
▪ Screenings considered easy to offer ▪ Screenings implemented with support from FUAM, FUNASA, and the coordinators of Alto Solimões DSEI ▪ Nurse technicians mostly indigenous and have a low turnover rate ▪ CHWs with previous experience in working with indigenous people ▪ Rapid testing not requiring laboratory infrastructure or highly trained health care workers ▪ Integration of screening activities into existing health services	▪ Difficulty in reaching remote areas and transporting RT and other consumables due to long distances and having only fluvial or air transport available ▪ Underperforming health services ▪ Need to maintain cold chain for rapid HIV testing ▪ Communication problems and cultural barriers between health care workers and indigenous people ▪ Lack of commitment of CHWs to screening indigenous people ▪ Need to screen adolescents due to early sexual initiation
Opportunities	Threats
▪ Brazilian Ministry of Health committed to screening programme ▪ Screening programme prioritised by FUNASA ▪ Rapid testing delivered by WHO and Ministry of Health using existing logistics ▪ Health services decentralised and free of charge ▪ Existing outreach activities to screen communities ▪ Funding available from Bill & Melinda Gates Foundation and the WHO	▪ Lack of trained health care workers ▪ Lack of treatment for syphilis- and HIV-positive individuals and lack of disposable protective products ▪ Uncertainty about testing acceptance by indigenous people ▪ Inconsistent support from DSEI coordinators ▪ Strikes

In the second phase, during initial training in the city of Tabatinga, the baseline questionnaire was administered to 41 health professionals involved in POC testing. This cluster was divided into twelve groups according to their polo base worksites. The semi-structured questionnaire included questions regarding demographics, vulnerability factors for HIV and syphilis among the indigenous people, and the operability, spatial accessibility, and performances of the health services. The groups were also asked to develop a screening strategy suitable for their own polo base and indigenous community.

Fourteen vulnerability factors for HIV and syphilis among indigenous communities in Brazil were selected [7]. These included gold mining as an income source; indigenous migration from rural to urban areas; having a DSEI located in a border area; facing restricted access to health services; inhabitation of an indigenous area with the presence of sex workers and other vulnerable groups, such as men who have sex with men, military personnel, religious groups, and non-indigenous foreigners; and polo base characteristics such as being located in a city, proximity to the city of Tabatinga (<1 h), being in a rural location close to an urban area (<1 h), proximity to the presence of narcotic traffic (<1 h), and high migration flow into indigenous communities. These components are considered cross-cutting issues that may influence the ways of life of indigenous communities at both the intraethnic and the interethnic levels, de-structure their social networks and their internal cohesion, undermine community responses [11], and increase their vulnerabilities to and risks of acquiring HIV and other STIs [12].

Data on spatial accessibility revealed the dimension of access to health care [13]. Accessibility was measured in terms of travel times via local transportation networks to health services sites (polo bases and health posts in each village). Participants mapped the villages in their catchment areas with concentric circles representing distances from the polo base and indicated the means of transport to the villages, specifying the power of the boat engine when applicable as well as the travel times to the villages. The same exercise was conducted to measure the distances from the polo bases to the DSEI head office in Tabatinga, which was used as a proxy for DSEI operability because all logistics depend on the head office. Participants were also asked about geographical and logistical barriers to spatial accessibility.

In the third phase, three months after the initiation of screenings in the field, health professionals answered a self-completed questionnaire with both closed and open questions to characterise the screening strategy adopted in each polo base and to evaluate the training received.

### Data analysis

#### Qualitative analysis

Qualitative data were analysed based on the grounded theory method [14]. The processing and coding of the narrative texts gathered from the interviews and focus groups followed the steps designed by Charmaz (2009), [15] which is a reinterpretation of the classic work of Glaser and Strauss. Charmaz specifically proposed a set of systematic actions that including selecting key words and main native concepts for the identification of meaningful social narratives from thorough analyses of speeches [15].

**Fig. 2:**
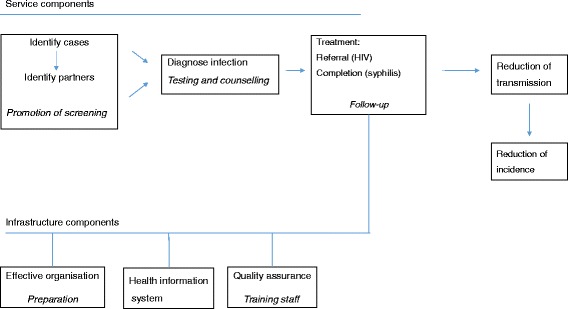
Logic model for the HIV- and syphilis-screening programme

Figure 2 presents the logic model for the implementation of HIV and syphilis screening. The thematic framework was developed based on the indexing and analysis of the collected data [16, 17]. Improvements in access to screening, treatments, quality assurance, and effective preparation were considered potential criteria for the future reduction in transmission of the infections. Six main activities relevant to routine programme operation were identified: at the infrastructure level, preparation, a health information system, and staff training, and at the service level, screening promotion, testing and counselling, and treatment and follow-up. Our hypothesis was that if service and infrastructure component activities took place, we would expect that infected patients with HIV or syphilis would be treated and linked to referral services, which would eventually reduce transmission of these infections. However, as we focused on the early implementation of the intervention, it was not possible to demonstrate an impact in terms of a reduction in the incidence. Instead, we collected a set of outcome measures that reflected the increase in access to screening, improved health outcomes (treated patients), acceptance of rapid testing by health professionals, quality assurance, and integration into existing programmes.

#### Quantitative analysis

We described categorical data using frequencies. A vulnerability score based on the 14 defined vulnerability components for HIV and syphilis was calculated for each polo base by assigning one point to each vulnerability dimension, with the exception of ‘restricted access to health care’, which could be assigned up to three points, depending on the number of barriers reported. Therefore, the maximum vulnerability score for each polo base was 16 points. For the mapping of the measure for the geographical distribution of health services in the Alto Solimões DSEI, we estimated the distances, measured as the travel times, between the DSEI head office and each polo base and between each polo base and its community. The distribution of villages in the catchment area of each polo base was classified into 4 time categories (<1 h, 1–2 h, 3–5 h, and > 9 h) to indicate the distance covered by boat between the villages and each polo base.

### Ethics

Ethical approval for this study was obtained from the Ethical Review Committee of Fundação Alfredo da Mata. All of the participants were informed about the research objectives before being interviewed or completing questionnaires, and all of them signed an informed consent form.

## Results and discussion

### Demographics and vulnerability to acquiring HIV and syphilis among the indigenous people

The Alto Solimões DSEI is located in the triple-border area and has 43,259 indigenous inhabitants, [18] 25,322 of whom are of reproductive age (58 %). The main spoken language is Ticuna; Portuguese is spoken by more than 50 % of the inhabitants in only seven of the twelve polo bases. The polo bases are coordinated [19] from the head office in Tabatinga, the capital of Alto Solimões. Each polo base team includes approximately nine health professionals and administrative staff members; only two polo base teams include a medical doctor. In general, nurses are non-indigenous and have a high turnover rate, whereas community health workers (CHWs) are indigenous and have a low turnover rate. CHWs perform a wide range of primary care and public health activities, including visiting households in their villages.

**Fig. 3:**
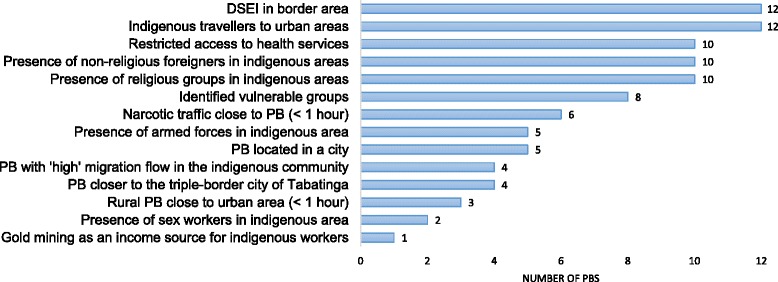
Vulnerability factors in the indigenous population

Overall, the DSEI earned a mean vulnerability score of eight (range: 6–14) (Fig. 3). The scores were higher for polo bases in the outskirts of Tabatinga due to the presence of the army, narcotics trafficking, and foreigners in indigenous areas. These are typical factors in border areas; [20] in fact, all of the polo bases cited their location in the border area as a vulnerability component. All of the polo bases also mentioned indigenous travel to urban areas as a vulnerability factor. Mobility is specifically considered to be a risk factor for HIV and syphilis infection [21]. Additionally, ten of the twelve polo bases mentioned restricted access to health services and the presence of foreigners in indigenous areas. Eight identified the existence of vulnerable groups, such as men who have sex with men, in their catchment area, which has been associated with increased transmission of HIV and STIs [22]. The vulnerability scores of urban areas were higher than those of rural areas.

The accessibility of indigenous communities constitutes a bottleneck for establishing health programmes [23–25]. Similarly, a study of indigenous people in Guatemala [23] identified geographical barriers and a lack of transport as the main limitations to accessing health care. POC testing for syphilis and HIV to enable immediate treatment has been shown to be a suitable alternative for the state of Amazonas and is likely to be effective in other similar settings [3, 26].

### Operability of the DSEI and its geographical and logistical accessibility to the indigenous communities

The locations and geographical distribution of the polo bases in relation to the DSEI and the distribution of the villages in the catchment area were among the determining factors in estimating both the vulnerabilities to acquiring HIV and syphilis and the accessibilities to the indigenous communities.

**Fig. 4:**
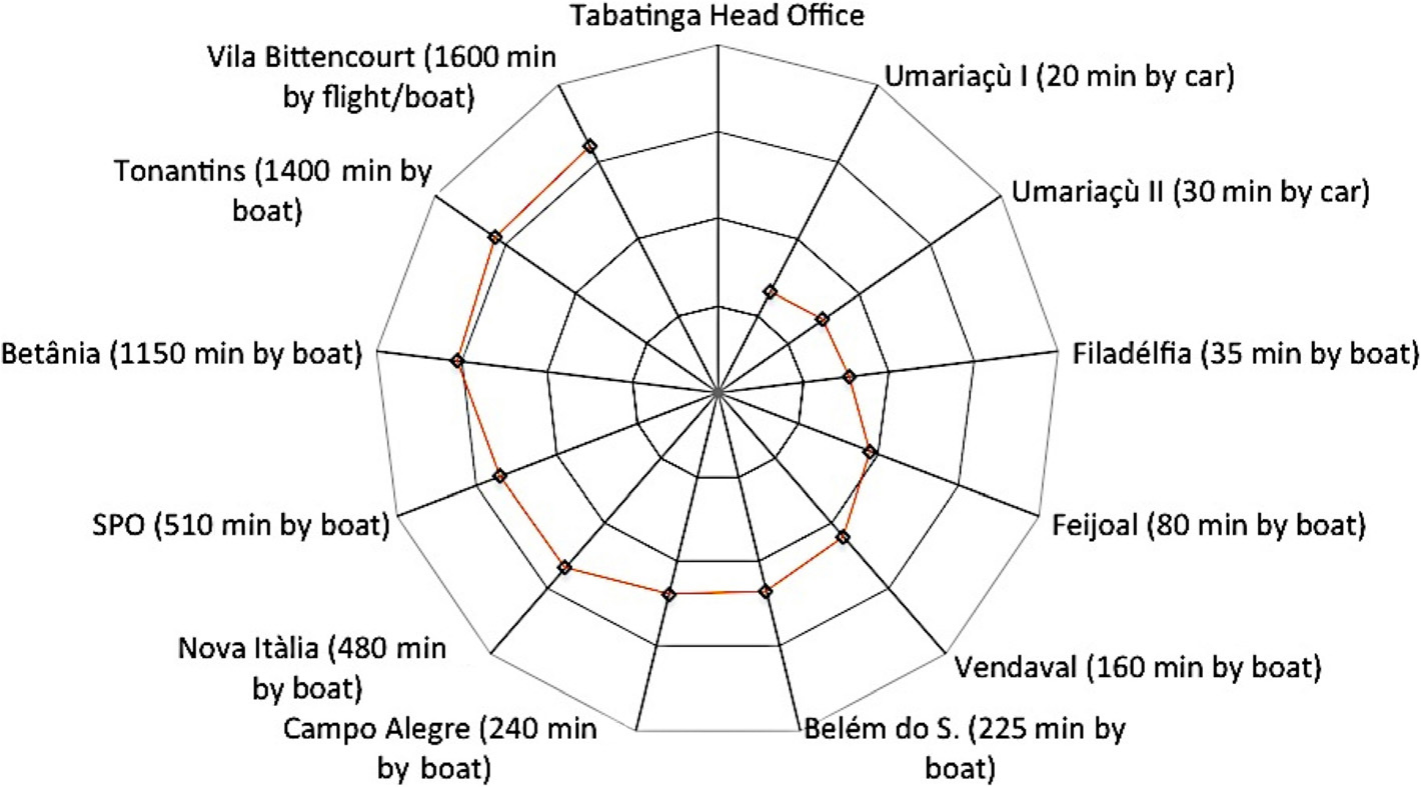
Distances (logarithmic scale) from the DSEI head office in Tabatinga to the polo bases, in minutes (60-hp engine)

Figure 4 displays the twelve polo bases distributed within a radius of 1600 min around the head office in Tabatinga, with the time standardised to a boat with a 60-hp engine. When we compared the distances to the polo bases using the real time needed, travel times ranged from 20 min to > 1 day and depended largely on the power of the boat’s engine. Most polo bases are only accessible by boat, although two are accessible by car, and one is only accessible by air. Four of the polo bases were classified as a long distance, or more than ten hours, from the city.

The number of villages belonging to one polo base ranges from 1 to 33. For nine (75 %) of the twelve polo bases, at least half of the villages are located at a distance of < 1 h from the primary health centre (PHC), and for three of the polo bases, the villages are located at distances ranging from 1 to 5 h from the PHC. Only one polo base has three villages (26 %) located at a distance requiring a three-day boat ride. Eight polo bases mentioned at least one geographical barrier (primarily no access during the dry season) and at least one logistical barrier, including a lack of gasoline (5 of 12), a lack of a boat engine (4 of 12), or a lack of a boat (3 of 12).

### Description and operability of health care services in the Alto Solimões DSEI

Most of the polo bases in Alto Solimões have electricity available 24 h per day. Adequate infrastructure was a critical step towards ensuring high-quality rapid testing and the administration of drugs and consumables because the screening materials require appropriate storage conditions.

The polo bases provide diagnostic services, primary care services (including antenatal care), and outreach visits, and the staff of most (7 of 12) visit their communities at least six times per year. Reported barriers to outreach visits included a lack of gasoline or a boat (7 of 12); geographical inaccessibility due to distance or the dry season, i.e., low rainfall from October to March (5 of 12); language and communication (5 of 12); and the interruption of health care services due to staff turnover (4 of 10).

### Performance of health services during the implementation of POC community testing for HIV and syphilis

Eleven polo bases implemented screening activities. Three months after the introduction, 6473 indigenous persons had been tested out of a total of 25,322 men and women of reproductive age (25.9 %) in the Alto Solimões DSEI. This number represents 15 % of the eligible population. A total of 165 syphilis cases were detected, representing an overall prevalence of 2.5 % (range: 0.0-4.5 %). Pregnant women represented 8.0 % of the individuals of reproductive age who were tested and 15 % of the detected syphilis cases, resulting in a prevalence of 1.2 % (range: 0.0-11.4 %). The prevalence of syphilis in pregnant women was previously estimated at 2.2 % by the antenatal services of the Alto Solimões DSEI [27]. Ten cases of HIV were also detected, with no cases detected in pregnant women.

### Preparation and organisation of screening activities

During the organisation of the screenings in the DSEI, collaboration between external consultants and governmental institutions was shown to be a successful strategy for achieving a sustainable health intervention [28]. Fundação Alfredo da Matta (FUAM), the screening initiator, brought field experience with the use of rapid testing [29] and, as a small institution, they had the flexibility to adapt to new situations. The DSEI head office in Tabatinga served as the indigenous health authority in the Alto Solimões DSEI. The operability of indigenous health services was directly related to the performance of the coordination and logistics of the Alto Solimões DSEI.

One of the perceived problems was the absenteeism of health professionals and the coordinator of the DSEI due to the tight schedule of the DSEI (continuing education, priority programmes, and health campaigns) and the high turnover of staff in remote areas, which has also been observed in other settings [30]. The absenteeism in particular contributed to an increased workload.

Most of the problems that negatively impacted the implementation of screening activities were related to logistics, and particularly the lack of availability of gasoline, which limited field visits (5 of 10 polo bases), and to stock-outs of tests and consumables, which limited the operability of the POC screening at the polo base level. This result emphasises the ‘threat’ identified in the SWOT analysis of the non-operability of the Alto Solimões DSEI. In this context, indigenous health professionals are advantageous for delivering POC screening because they have very low turnover and speak the local language.

### Preparation for the screening activities in the polo bases

During the preparation for the screening activities in the polo bases, all ten teams of health professionals organised various team meetings and training sessions with CHWs to adjust the screening strategy. On the second questionnaire, most of these teams (9 of 10) reported that they had experienced no challenges in motivating their CHWs. However, 6 of 10 had to train additional health professionals to fulfil the screening objectives set by the DSEI. As part of the screening implementation training, six polo bases included additional training activities to address translation problems and three polo bases included additional training on patient confidentiality. CHWs are crucial because they serve as an entry point to indigenous communities, and the majority of the polo bases (9 of 10) rated their collaboration with CHWs as excellent or good.

Screening activities were performed at the polo bases and during field visits. For all of the polo bases, the optimal method for offering POC screening was to integrate it into routine consultations. In addition, five out of ten of the polo bases organised special screening days at the polo bases and priority interventions during outreach work, either in combination with EPI or solely for screening.

### Promotion of the screening activities in the indigenous communities

Screening activities in the indigenous communities were carried out at polo bases and schools. On the second questionnaire, health professionals reported that the involvement of CHWs, community leaders and teachers in promotion activities was a successful strategy. Informational materials were distributed but were not available in indigenous languages at most polo bases. In nine out of ten polo bases, language barriers were not considered to be a problem because the team was assisted by an indigenous health professional, a CHW or teachers.

### Testing and counselling

In the evaluation of POC testing in the field, health professionals did not report technical problems in the handling of rapid testing, and the fact sheets were considered practical tools. However, difficulties with fingerpricks were observed among health professionals not trained by FUAM. Health professional teams specifically reported that the acceptance of rapid testing was excellent or good at all ten polo bases, although two of them had concerns about the pain caused by the fingerpricks. Counselling was performed by nurses, and health professionals at five of the ten polo bases considered the communication of positive results to be challenging; this finding was determined via an open question. Through yes/no questions, it was determined that information provided through counselling was considered to be accurate and complete, with the exceptions of safe sex messages (2 of 10) and information about follow-up testing (2 of 10). Through questions with Likert-like scale responses, it was found that health professionals at nine of the polo bases did not consider language and cultural barriers to accessing health services to be problems [23], and six polo bases had trained health professionals or CHWs who offered counselling or who performed simultaneous translation. Additionally, workers only considered patient confidentiality to be highly important at five polo bases, which is a major concern for community-wide screening events because this issue poses an increased threat to patient privacy and confidentiality.

### Follow-ups and case notifications

Evaluations of treatment adherence and referrals revealed that 86.7 % of all syphilis-positive individuals started treatment following the rapid test and that all were referred for confirmatory testing. Failure to immediately treat syphilis-positive individuals was primarily caused by a lack of benzathine benzylpenicillin at four of the ten polo bases. All HIV-positive individuals were referred to specialised HIV/AIDS clinics in the cities of Tabatinga or Benjamin Constant according to standard procedures. All of the syphilis- and HIV-positive individuals referred their partners for testing, which was a very promising result, as evidence has revealed that more than half of index patients in developing countries fail to refer their partners [31].

All of the polo bases considered the case reporting sheets to be easy to complete. However, nine of the ten were not aware of the compulsory notification of HIV and syphilis cases in pregnant women or of congenital syphilis and mother-to-child HIV transmission, even after receiving training. Mandatory reporting should thus be emphasised during training, and official case notification forms should be distributed to improve the health information system.

### Training performance

The trainings organised by the Alfredo da Matta Institute in Manaus as well as in the reference laboratory in Tabatinga were both evaluated using a second self-completed questionnaire. Training was considered to be appropriate by the health professionals at eight of the ten polo bases, and all of the polo bases considered the hand-outs and materials to be easy to use. Field observations during the training revealed that the trainees had trouble understanding treatment regimens, storage conditions, and the communication of positive test results. In understaffed settings with high turnover, a possible strategy to offset this challenge would be to expand the initial training in order to establish a larger pool of trained staff, creating redundancy and allowing other staff members to substitute for those who leave. Table 2 presents a summary of the enabling and hindering factors in the implementation strategy for syphilis and HIV screening among the indigenous people in the present study.

**Table 2:** Enabling and hindering factors in the implementation of syphilis and HIV POC screening among the indigenous people in the Alto Solimões DSEI

Main categories	Enabling factors	Hindering factors
Preparation	- Collaboration of the main actors and well-defined roles and responsibilities - Reliable supply chain (FUAM) - Organised transport to the field - Planning of screening activities with the collaboration of CHWs and consideration of other scheduled work at the polo base	- Absenteeism of health professionals due to full schedule of health activities and interventions in the DSEI - Insufficient supply of drugs and consumables - Logistics at DSEI head office in Tabatinga (shortage of gasoline) and transportation logistics of FUNASA Manaus
Promotion of screening activities in the indigenous population	- Strategies to promote activities in indigenous communities include: - Contact: CHWs, village leaders, teachers - Population: area of 1 CHW - Sites: polo bases, indigenous communities - Topics: health education, screening activities - Translators: CHWs, teachers	- Lack of IEC material
Testing	- Availability of rapid testing assured - Technical functionality of rapid testing used for the present screening - Organisation of the cold chain during field visits (e.g., combined with vaccination)	- Insufficient number of trained health professionals - Incorrect handling of rapid testing (fingerprick) - Insufficient understanding of the necessity of syphilis testing - Acceptance of rapid testing in indigenous communities limited because of anxiety about the pain of testing and possibility of a positive result
Counselling	- Guarantee of the accuracy of the translators collaborating with the skilled health professionals and CHWs - Guarantee of patient confidentiality - Inclusion of translations and patient confidentiality in the preparation meetings and trainings for the polo base team - Consistent partner notification	- Language not adapted to the indigenous population - Incomplete and incoherent information during counselling - Insufficient patient understanding of the importance of being tested for syphilis - Insufficient ability to address patient anxiety - Lack of respect for patient confidentiality - Lack of privacy during counselling
Follow-up	- Organisation of medication administration (1st dose immediately after test; CHW must schedule and bring patient to a polo base for 2nd and 3rd doses) - Organisation of follow-up in the DSEI	- Insufficient stock of benzathine benzylpenicillin
Health information system	- Monitoring sheet clear and easy to handle - Data processing performed by epidemiology team at FUAM	- Compulsory notification not completed - Delayed collection of the monitoring data (FUNASA transportation logistics)
Training of the HP on the screening activities (FUAM)	- Collection of required data regarding the study site (DSEI, health professionals) and screening population - Efficient organisation of the training (identification of the number of health professionals to be trained and organisation of the infrastructure) - Development of hand-outs and materials regarding the performance of screening using rapid testing in the field	- Inappropriate language for indigenous people (too technical) - Inappropriate hand-outs - Practical part not adapted to field conditions (only applicable to laboratory conditions) - No uniformity in the training curriculum (various trainers) - Technical and counselling contents are inconsistent - No consideration given to the suggestions of the health professionals regarding the adaptation and improvement of training (assessment of the training) - Insufficient focus on problems related to the communication of a positive test result

*CHW* community health worker, *DSEI* Special Indigenous Health District, *FUAM* Fundação Alfredo da Matta, *IEC* information, education, and communication

## Conclusions

In this study, both locations in the border area and mobility were consistently acknowledged as vulnerability factors. Contextually appropriate approaches that address these factors might stand a greater chance of long-term success in controlling HIV and syphilis in this region. Only two polo bases had no geographical restrictions in access to their villages, and for areas with geographical and transportation barriers to health care access, POC testing is a helpful approach. The study results form the basis for the design of strategies to improve the feasibility, viability, and sustainability of introducing HIV and syphilis POC testing on a larger scale in nine DSEIs in the Amazon region. Recommendations include addressing the preparation phase at the coordination and training levels to impact the overall implementation in the field.

A limitation of this study was the use of non-random samples, which affects the generalisability of the results. Presenting vulnerability components as 14 ‘drivers’ of HIV and syphilis may also have missed several key issues resulting from the complex nature of vulnerability.

Lessons learnt in this remote region could be applied in other settings characterized by operational challenges and vulnerability factors in order to overcome barriers to the introduction of testing. In settings with few financial resources, collaborations between external consultants and governmental institutions facilitate sustainable public health interventions. Tools such as checklists and fact sheets are very simple methods to facilitate this process and to provide high-quality information in a timely manner. A list could be especially useful at both the coordination and polo base levels for guiding the completion of the administrative and logistical prerequisites of implementation strategies. Moreover, SWOT analyses give a rapid overview of an entire intervention and improve preparedness for upcoming threats. To promote POC screening activities and counselling, we propose that strategies aimed at enhancing patient confidentiality and the accuracy of translations be integrated into the information and training sessions for the polo base teams and CHWs. It would also be beneficial to generate culturally sensitive information, education, and communication (IEC) materials on health topics to improve the ease of communication; a lack of access to health information is often one of the many barriers to better health [32]. Training should be based on the following principles: the simplification of technical language, the adaptation of content to the health workers’ levels of knowledge of the indigenous context, the inclusion of case studies or questions and difficulties raised by participants to increase the relevance of the training to the field; and the creation of fact sheets and protocols for syphilis testing, treatment, follow-up and counselling to improve communication between stakeholders. Based on this recommendation, the implementation of POC screening in nine DSEIs over three years included the development of flipcharts and fact sheets for syphilis counselling, which were adapted to the local contexts and implemented in the field as back-up materials.

The development of monitoring sheets for POC screening for HIV and syphilis, including nationally and internationally comparable indicators, as well as the reinforcement of compulsory notifications can contribute to the strengthening of the health information system.

## References

[1] Benzaken AS, Pinto NV, Carvalho CH, Peeling RW (2011). Increasing access to HIV and syphilis screening in remote areas using rapid tests. Symposium 2: Rapid tests as tools to transform policy, strengthen health systems and save lives; 19th Biennial Conference of The International Society For Sexually Transmitted Diseases Research; 2011 July 10–13. City of Quebec, Quebec. Sex Transm Infect.

[2] Benzaken A, Sabido M, Galban E, Rodrigues Dutra DL, Leturiondo AL, Mayaud P (2012). HIV and sexually transmitted infections at the borderlands: situational analysis of sexual health in the Brazilian Amazon. Sex Transm Infect.

[3] Stoner BP (2008). Rapid tests for maternal syphilis screening: effective and cost-effective. Sex Transm Dis.

[4] World Health Organization. Department of Reproductive Health and Research (2007). The global elimination of congenital syphilis: rationale and strategy for action.

[5] Joint United Nations Programme on HIV/AIDS (UNAIDS) (2012). Treatment 2015.

[6] Mabey DC, Sollis KA, Kelly HA, Benzaken AS, Bitarakwate E, Changalucha J (2012). Point-of-care tests to strengthen health systems and save newborn lives: the case of syphilis. PLoS Med.

[7] Ministério da Saúde (2005). Secretaria de Vigilância em Saúde. Programa Nacional de DST e Aids. Distritos Sanitários Especiais Indígenas Diretrizes para implantar o Programa de DST/AIDS.

[8] Athias R, Machado M (2001). Indigenous peoples’ health and the implementation of Health Districts in Brazil: critical issues and proposals for a transdisciplinary dialogue. Cad Saude Publica.

[9] Joint United Nations Programme on HIV/AIDS (UNAIDS) (1998). Guide to the strategic planning process for a national response to HIV/AIDS.

[10] O’Cathain A, Thomas KJ (2004). “Any other comments?” Open questions on questionnaires - a bane or a bonus to research?. BMC Med Res Methodol.

[11] Hita SR (2014). Health, globalization and interculturalism: an anthropological approach to the situation of indigenous peoples in South America. Cien Saude Colet.

[12] Orellana ER, Alva IE, Cárcamo CP, García PJ (2013). Structural factors that increase HIV/STI vulnerability among indigenous people in the Peruvian amazon. Qual Health Res.

[13] Guagliardo MF (2004). Spatial accessibility of primary care: concepts, methods and challenges. Int J Health Geogr.

[14] Strauss A, Corbin J (2008). Pesquisa Qualitativa Técnicas e procedimentos para o desenvolvimento da Teoria Fundamentada.

[15] Charmaz K (2009). A Construção da Teoria Fundamentada. Guia Prático para Análise Qualitativa.

[16] Pope C, Ziebland S, Mays N (2000). Qualitative research in health care. Analysing qualitative data. BMJ.

[17] Adamson J, Gooberman-Hill R, Woolhead G, Donovan J (2004). ‘Questerviews’: using questionnaires in qualitative interviews as a method of integrating qualitative and quantitative health services research. J Health Serv Res Policy.

[18] Instituto Brasileiro de Geografia e Estadística (IBGE). Sinopse do Censo Demográfico 2010. Tabela 1.4 - População nos Censos Demográficos, segundo as Grandes Regiões e as Unidades da Federação - 1872/2010. Available at: <http://www.ibge.gov.br/home/estatistica/populacao/censo2010/tabelas_pdf/Brasil_tab_1_4.pdf>. Last accessed: 16 May 2013.

[19] Ministério da Saúde (2011). SIS Fronteiras: Sistema Integrado de Saùde das Fronteiras 2005: Integração das ações de saúde na fronteira.

[20] Peiter PC (2007). [Living conditions, health status and health services availability along the Brazilian border: a geographical approach]. Cad Saude Publica.

[21] Napolitano DA (2007). Towards Understanding the Health Vulnerability of Indigenous Peoples Living in Voluntary Isolation in the Amazon Rainforest: Experiences from the Kugapakori Nahua Reserve, Peru. EcoHealth.

[22] Szwarcwald CL, Barbosa-Junior A, Pascom AR, de Souza-Junior PR (2005). Knowledge, practices and behaviours related to HIV transmission among the Brazilian population in the 15–54 years age group, 2004. Aids.

[23] Hautecoeur M, Zunzunegui MV, Vissandjee B (2007). Barriers to accessing health care services for the indigenous population in Rabinal, Guatemala. Salud Publica Mex.

[24] Grenfell P (2008). Anaemia and malaria in Yanomami communities with differing access to healthcare. Trans R Soc Trop Med Hyg.

[25] Peiris D, Brown A, Cass A (2008). Addressing inequities in access to quality health care for indigenous people. CMAJ.

[26] Tucker JD, Bu J, Brown LB, Yin YP, Chen XS, Cohen MS (2007). Accelerating worldwide syphilis screening through rapid testing: a systematic review. Lancet Infect Dis.

[27] Benzaken AS, Sabido M, Galban E, Pedroza V, Araújo AJ, Peeling RW (2011). Field performance of a rapid point-of-care diagnostic test for antenatal syphilis screening in the Amazon region, Brazil. Int J Std Aids.

[28] Huppatz C (2008). “Sorry” - in word and actions. Improving health in rural and remote Indigenous communities. Rural and Remote Health.

[29] Benzaken AS, Sabido M, Galban EG, Pedroza V, Vasquez F, Araújo A (2008). Field evaluation of the performance and testing costs of a rapid point-of-care test for syphilis in a red-light district of Manaus, Brazil. Sex Transm Infect.

[30] Schoo AM, Stagnitti KE, Mercer C, Dunbar J (2005). A conceptual model for recruitment and retention: allied health workforce enhancement in Western Victoria, Australia. Rural Remote Health.

[31] Alam N, Chamot E, Vermund SH, Streatfield K, Kristensen S (2010). Partner notification for sexually transmitted infections in developing countries: a systematic review. BMC Public Health.

[32] Shahid S, Finn L, Bessarab D, Thompson SC (2009). Understanding, beliefs and perspectives of Aboriginal people in Western Australia about cancer and its impact on access to cancer services. BMC Health Serv Res.

